# Leguminous allergy in a sample of children attending an outpatient allergy clinic

**DOI:** 10.1186/2045-7022-5-S3-P67

**Published:** 2015-03-30

**Authors:** Joana Belo, Elena Finelli, Joana Extreia, Pedro Martins, Paula Leiria-Pinto

**Affiliations:** 1 Hospital Dona Estefania, Centro Hospitalar Lisboa Central, EPE, Lisbon, Portugal; 2 Hospital Nossa Senhora do Rosario, Centro Hospitalar Barreiro Montijo, EPE, Barreiro, Portugal

## Background

An average of 4 Kg of leguminous per habitant per year is consumed by the Portuguese population. The most consumed leguminous are the beans followed by the chickpeas. There is a paucity of information available regarding the allergy to these leguminous.

## Objectives

To characterize the clinical manifestations of leguminous allergy in a Portuguese pediatric sample.

## Methods

The clinical records of six children with a history of leguminous allergy were retrospectively analyzed. The criteria of leguminous allergy was based on a reliable clinical history supported by complementary diagnostic tests, namely skin prick tests (SPT) and/or serum specific IgE.

## Results

The median age of onset of the allergic reaction was 3.9 ±2.2 years. The leguminous most commonly responsible for allergy were the peas - involved in 5 reactions, followed by chickpeas and beans, each involved in three reactions. All children were allergic to more than one leguminous, with a median of two leguminous involved in allergic reactions. All reactions except for one were immediate with a mild to severe degree of severity. Regarding the clinical findings of the allergic reactions, the mucocutaneous, gastrointestinal and respiratory symptoms occurred with similar frequency. Oral allergy syndrome was seen in one reaction. Two of these reactions fulfilled anaphylaxis criteria. All children except for one were allergic to at least another class of food besides the leguminous. Concomitant allergic rhinitis was also seen in all patients except for one. SPT, namely prick to prick tests, were performed and confirmed the allergy in all patients.

## Conclusion

In this sample, peas and chickpeas were responsible for most of the allergic reactions. The clinical features were variable and ranged from mild to severe. All children were allergic to more than one leguminous. The majority of the patients had multiple food allergy. The most common comorbidity was allergic rhinitis. The prick to prick skin tests seem to have a reliable clinical association regarding the diagnosis of leguminous allergy.

